# Comparing parental distress and children’s difficulties between parents of children with rheumatic diseases and parents of healthy children in families facing the COVID-19 pandemic

**DOI:** 10.1007/s12144-022-03589-8

**Published:** 2022-08-06

**Authors:** Sonia M. Bramanti, Valerio Manippa, Alessandra Babore, Anna Dilillo, Alessia Marcellino, Vanessa Martucci, Saverio Mallardo, Sara Isoldi, Silvia Bloise, Mariateresa Sanseviero, Donatella Iorfida, Enrica De Luca, Carmen Trumello, Francesca D’Alleva, Flavia Ventriglia, Riccardo Lubrano, Emanuela Del Giudice

**Affiliations:** 1 Department of Psychological, Health and Territorial Sciences, University “G. d’Annunzio” of Chieti-Pescara, Via dei Vestini, 31, 66100 Chieti, Italy; 2 Department of Education, Psychology and Communication, University of Bari Aldo Moro, Bari, Italy; 3 Pediatric and Neonatology Unit, Maternal and Child Department, Sapienza University of Rome, Polo Pontino, Latina, Italy

**Keywords:** Parent psychosocial functioning, COVID-19, School-age children, Hyperactivity and ADHD, Chronic and recurrent pain, Rheumatology

## Abstract

The COVID-19 pandemic could be a threat for the health status of children with a chronic condition. The present study aimed to explore parents’ and children’s psychological adjustment during the current pandemic, pursuing a triple objective: to compare the psychological adjustment of parents of children with pediatric rheumatic diseases (PRDs) and parents of healthy children; to analyze children’s psychological symptoms (emotional problems and hyperactivity) before and during the COVID-19 pandemic, and with or without a PRDs diagnosis; to explore the associations of children’s emotional problems and hyperactivity with parents’ psychological adjustment, parent–child interactions and belonging or not to families with PRDs. This cross-sectional study involved 56 parents of children with PRDs and 53 parents of healthy children. Self-report questionnaires about parents’ depression, anxiety, parenting stress, and children’s emotional symptoms and hyperactivity-inattention were administered. No differences were detected on psychological adjustment between parents of children with PRDs and parents of healthy children. Parents of children with PRDs reported statistically significant higher levels of children’s emotional problems and hyperactivity before the pandemic, compared to parents of healthy children; during COVID-19 pandemic, emotional symptoms increased for both groups, while hyperactivity-inattention symptoms increased only in the group of healthy children. Children’s emotional difficulties were associated with higher levels of parental anxiety, worse parent–child interaction and having PRDs; children’s hyperactivity symptoms were related to parent–child difficult interaction and higher levels of parental depression. Findings suggest the importance to target the children in relation to their parents, when approaching the psychological aspects of PRDs.

## Introduction

The diagnosis of a chronic illness in children is upsetting not only for children themselves but also for their parents as it often implies facing several changes in daily routines and challenges. These last include the management of little patients’ pain, fatigue, problems derived from school absenteeism, hospitalizations and treatments (Fortuna-Reyna et al., 2021).

Among the chronic conditions, pediatric rheumatic diseases (PRDs) are a heterogeneous group of disorders associated with abnormal immune system functioning, starting in childhood and often persisting into adulthood. Children with PRDs could have a disabling and impairing quality of life, despite substantial progress in disease management over the years (Cohen et al., 2017; Torres-Made et al., 2020). Juvenile idiopathic arthritis is the most common chronic rheumatic disease in childhood with a prevalence of approximately 1 to 2 per 1000 children, and an incidence of 11 to 14 new cases per 100000 children (Del Giudice et al., 2017; Prakken et al., 2011). Beyond the physical impairments, existing studies have shown that children with PRDs experience higher levels of psychological difficulties, as compared with healthy children (Noll et al., 2000; Wagner et al., 2003).

Previous research has mainly focused on psychological implications for children, neglecting parents and their psychological reactions as also noticed by Gómez-Ramírez et al. (2016). This lack of studies is surprising, even considering that parents’ psychological reactions and experiences related to the offspring’s illness are associated with children’s disease outcomes (Knafl et al., 2015; Ryan et al., 2013). In fact, as a practical matter, adherence to PRDs treatment requires the collaboration of parents who are responsible for children’s health practices and attendance at medical appointments (Gómez-Ramírez et al., 2016; Jones et al., 2009). Furthermore, previous studies carried out on other childhood chronic conditions consistently highlighted associations between parental adjustment and patients’ outcomes (Honey & Halse, 2006; Nelson et al., 2012; Woodgate et al., 2008). As for the specific topic of PRDs, Wagner et al. (2003) highlighted that the higher the parent distress, the greater the children’s depression.

Parents’ mental health problems may affect children’s adjustment, mainly during such stressful events as the current pandemic (Courtney, 2020; Bıkmazer et al., 2020; Morelli et al., 2022). Since the end of December 2019, the Coronavirus Disease (COVID-19) has spread around the world, to the point of being defined as a pandemic (World Health Organization; WHO, 2020), which caused a high number of infections and deaths. Given these premises, it is understandable that families with children with PRDs may feel particularly at risk, with possible psychological implications, such as an increase in distress and emotional problems levels for both children and parents.

In spite of this, studies on the psychological implication of COVID-19 pandemic on children with PRDs and their parents are still scarce. For instance, Durcan et al. (2021) found higher distress in parents of children with PRDs than in parents of healthy children. This result was not confirmed in parents of children with PRDs younger than 12 years. However, in their study, they did not deepen neither the child-parent interaction nor how parents perceived their children’s emotional and behavioral symptoms. With regard to other chronic diseases in children, data on the psychological effects of the COVID 19 pandemic on parents are still limited and controversial, showing for example a higher anxiety in mothers of patients with cystic fibrosis (Pınar Senkalfa et al., 2020) but not in parents of lung disease children (Ademhan Tural et al., 2020). A recent study found that caregivers of children with special healthcare needs exhibited more emotional distress than parents of children without special healthcare needs (Liu et al., 2021).

According to these premises, our first aim was to detect differences in parents’ psychological adjustment (with regard to anxiety, depression and anxiety associated with COVID-19) and parent–child interactions between parents of children with PRDs (i.e., clinical group; CL) and parents of healthy children (i.e., healthy control group; HC). We expected higher symptomatology in the CL group than in HC group.

A second aim was to analyze parents’ perception of children’s psychological symptoms (emotional problems and hyperactivity) before and during the COVID-19 pandemic, also considering the presence or not of a PRDs diagnosis. Given the inconsistent findings of existing research on this theme, hypotheses could not be formulated on the direction of these differences. Finally, we explored the associations of parents’ psychological adjustment, parent–child interactions and belonging to the CL or HC group with children’s emotional problems and hyperactivity. We expected a positive association between parents’ anxiety and depression and children’s maladjustment.

## Participants and procedure

Two a priori power analyses were conducted using the software developed by Soper (Soper, 2022), to determine the recommended minimum sample size for testing differences on one independent variable with two groups and conducting multiple regression. A moderated effect size of 0.5 was anticipated with power level set at 0.80 and a significant alpha level set at 0.05. The minimum total sample size necessary to detect significant differences between groups was *N* = 102, 51 participants for each group. As regards multiple regression, it was considered a moderated effect size of 0.15 was anticipated with power level set at 0.80, a significant alpha level set at 0.05 and five predictors. The required minimum sample size to run a regression analysis and detect a significant effect was *N* = 91.

The present study was conducted in Italy via an online survey in May–June 2021, during the last period of COVID-19 lockdown restrictions. The total sample (*N* = 109) was composed of Italian parents (*N* = 109, 79 mothers and 30 fathers) aged from 31 to 60 years old (*M* = 45.9, *SD* = 6.1). Specifically, the clinical group (CL) comprised 56 parents of 40 children affected by PRDs recruited among those who regularly visited the Pediatric Unit of Santa Maria Goretti Hospital, Latina—Sapienza University of Rome (Polo Pontino), whereas the healthy control (HC) group was composed of 53 parents of 42 children without any chronic disease, recruited using snowball type of sampling.

Participants were informed about the purpose and procedure of the study, and that their participation would be anonymous, voluntary, and uncompensated on the first page of the survey, and each gave his/her consent for participation by clicking “Yes, I accept to participate in the study”. All the tools were administered with a single administration. All procedures and questionnaires assessed were fully compliant with the Declaration of Helsinki, with the American Psychological Association Ethical Principles and with the Ethics Code of the Italian Board of Psychology (the regulatory Authority providing the national guidelines for research and clinical practice). This study was approved by the Institutional Review Board of Psychology of the Department of Psychological, Health and Territorial Sciences of the University “G. d'Annunzio” (Chieti)  (Protocol Number 21011).

## Measures

### Socio-demographic and medical screening characteristics

We created an ad hoc questionnaire to explore socio-demographic data to assess parents’ general information (e.g., age, gender, education, employment status) and child’s characteristics (gender and age). Clinical features such as disease type, duration and treatment were assessed by the pediatric rheumatologist of the pediatric hospital ward, who usually follows them. All these data are reported in Table 1.

**Table 1:** Parents’ and children’s socio-demographic and clinical characteristics

Parents’ socio-demographic characteristics	Clinical group (*N* = 56)	Healthy control group (*N* = 53)	Comparisons by group
Age (M years ± SD)	45.61 ± 5.74	46.15 ± 6.50	t = 0.87
Gender			
Male	17	13	χ^2^_1_ = 0.53
Female	39	40	χ^2^_1_ = 0.01
Marital status			
Married	44	40	χ^2^_1_ = 0.19
Unmarried	12	13	χ^2^_1_ = 0.40
Education degree			
Middle School	23	12	χ^2^_1_ = 3.46
High School	22	26	χ^2^_1_ = 0.33
University	8	11	χ^2^_1_ = 0.47
Post-University	3	4	χ^2^_1_ = 0.14
Occupation			
Employed	40	45	χ^2^_1_ = 0.29
Unemployed	16	8	χ^2^_1_ = 2.67
Household income			
< 28000 €	47	29	χ^2^_1_ = 4.26 (*p* = .039)
> 28000 €	9	24	χ^2^_1_ = 6.82 (*p* = .009)
Household income during pandemic			
Unvaried	27	32	χ^2^_1_ = 0.42
Improved	2	4	χ^2^_1_ = 0.20
Worsened	27	17	χ^2^_1_ = 1.04
Familiar relationship during pandemic			
Unvaried	22	24	χ^2^_1_ = 0.09
Improved	20	17	χ^2^_1_ = 0.71
Worsened	14	14	χ^2^_1_ = 0.00
Children’s age (M years ± SD)	13.21 ± 5.54	12.13 ± 3.86	t = -1.07
Children’s gender			
Male	19	23	χ^2^_1_ = 0.38
Female	21	20	χ^2^_1_ = 0.10
Children’s PRD diagnosis			
Juvenile Idiopathic Arthritis	25		
Autoinflammatory Conditions	9		
Other PRDs	6		
Children’s PRD duration (in years)	6.57 ± 4.260		
Children in treatment	**27**		

### Questionnaires

The Italian version (Annunziata et al., 2011) of the Hospital Anxiety and Depression Scale (HADS; Zigmond & Snaith, 1983) was used to assess both anxiety and depression symptoms of our sample. HADS is a brief self-reported questionnaire consisting of 14 items (sample items: “Worrying thoughts go through my mind”, “I feel cheerful”) in which participants are asked to rate how they felt during the previous week using a 4-point Likert scale. For each subscale, namely anxiety and depression, a subscore was ranging from 0 to 21. Higher score indicates higher levels of depressive and anxiety symptoms. Cronbach’s alphas for anxiety and depression scales were respectively 0.85 and 0.72.

The Italian version (Mozzoni & Franzot, 2020) of the Coronavirus Anxiety Scale (CAS; Lee, 2020) was used to assess the COVID-19 related anxiety in the two last weeks. CAS is a 5 items self-reported questionnaire (sample item: “I felt dizzy, lightheaded, or faint, when I read or listened to news about the coronavirus”) employing a 5-point Likert scale ranging from 0 (“never”) to 4 (“almost every day”). The level of anxiety was measured by summing the item responses, ranging from 0 to 20. A score equal to or greater than 9 indicates dysfunctional COVID-19 related anxiety. Cronbach’s alpha was 0.79.

The Parenting Stress Index – Short Form (Abidin, 1990; Italian version by Guarino et al., 2008), a self-reported tool designed to assess the overall level of stress experienced by parents, has three subscales. For our aims, we used only the Parent–Child Dysfunctional Interaction (P-CDI) scale, consisting of 12 items (sample item: “My child rarely does things for me that make me feel very good”). Participants are asked to rate their agreement on a 5-point Likert scale ranging from 1 (“strongly agree”) to 5 (“strongly disagree”). Higher scores indicate higher levels of parent–child dysfunctional interaction. In the present study, Cronbach’s alpha for P-CDI scale was 0.89.

The Strengths and Difficulties Questionnaire (SDQ; Goodman, 1997; Italian version by Tobia & Marzocchi, 2018) is a brief emotional and behavioral screening tool for children that can be completed by parents or teachers. For our aims, we selected the following two subscales: the “emotional symptoms” (sample item: “Many worries, often seems worried”) and “hyperactivity-inattention” (sample item: “Easily distracted, concentration wanders”). They are made up of 5 statements evaluated on a 3-point Likert scale ranging from 0 (“not true”) to 2 (“certainly true”). In the present study, both scales were administered twice: once in which parents are asked to respond referring to the last two weeks and the second one in which parents are asked to answer referring to the period before the pandemic (before March 2020). For each version and subscale, responses are summed to yield four different scores (emotional symptoms before pandemic; emotional symptoms during pandemic; hyperactivity-inattention before pandemic; hyperactivity-inattention during pandemic). Scores of each scale range from 0 to 10, with a higher score indicating higher parents’ perception of emotional and hyperactivity-inattention symptoms in their child. Cronbach’s alphas for the current and the past form were respectively 0.80 and 0.78.

## Data analysis

We conducted statistical analyses with IBM Statistical Package for Social Sciences (SPSS, version 19) software, significance level for all analyses were set at *p* < 0.05. First, we check the distribution of all variables by calculating their values of skewness and kurtosis. Variable distributions were found to be acceptably normal, as their range were between − 1 and + 1.

Then, we performed a series of student t-tests by group to compare anxiety, depression, COVID-19 related anxiety and parent–child difficult interaction scores of CL and HC parents.

Also, to compare parents’ perceptions of the child’s emotional symptoms and hyperactivity-inattention before and during COVID-19 pandemic, we conducted a 2 × 2 analysis of variance (ANOVA) for each dependent variable (emotional symptoms and hyperactivity-inattention scores) using as between factor the Group (CL vs. HC) and as within factor the Period (Before vs. During the pandemic). Tuckey post-hoc analysis was applied when a significant interaction was found.

Finally, we performed a series of linear regression analyses to test the effects of anxiety and depression, COVID-19 related anxiety, parent–child difficult interaction and having a child with PRDs on parent perception of child’s emotional symptoms and hyperactivity-inattention. In the regression model, parents were coded as a dummy variable (1 = having a child with PRDs; 0 = having a healthy child).

## Results

No differences were detected in anxiety and depression scores, COVID-19 related anxiety, and parent–child difficult interactions between CL parents and HC parents. Descriptive statistics and comparisons between the two groups are summarized in Table 2.

**Table 2:** Scores reported by our samples to the HADS (assessing anxiety and depression), CAS and P-CDI

	Clinical group	Healthy control group	
*Questionnaire scores*	*M* ± *SD*	*M* ± *SD*	*Student t-test by group*
Anxiety (HADS)	7.38 ± 4.76	7.21 ± 4.22	t = -0.19
Depression (HADS)	7.32 ± 4.22	6.19 ± 3.63	t = -1.50
COVID-19 anxiety (CAS)	2.98 ± 3.35	3.34 ± 3.42	t = 0.23
Parent–child difficult interaction (P-CDI)	25.41 ± 9.33	25.40 ± 9.48	t = 0.02

**Fig. 1:**
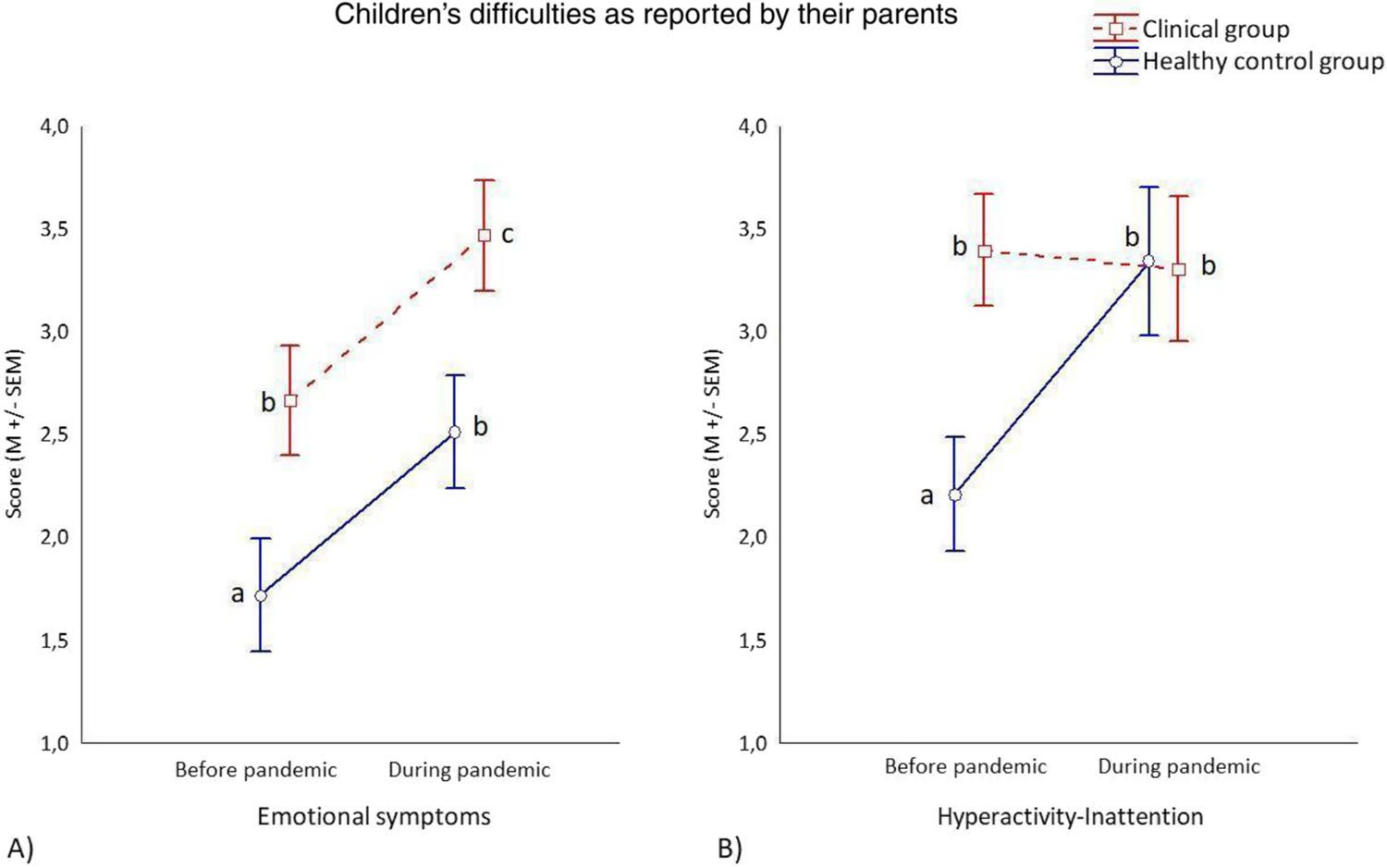
The 2 X 2 ANOVAs performed on emotional symptoms (1A) and hyperactivity-inattention (1B) scores (Mean ± Standard Errors of Mean). Note. On the left side (Fig. 1A) the graph underlies the main effects of the Group (higher scores for CL) and of the Period (higher scores during pandemic) with no interaction. On the right side (Fig. 1B) the graph underlies the main effect of the Period (higher scores during the pandemic) and the interaction Group X Period, with the HC group reported an increase of their child’s hyperactivity-inattention difficult during the pandemic and a lower difficult compared the CL sample but only before the pandemic. Different letters on the graphs indicate significant comparisons (*p* < . 05)

As regard the parent-reported child’s emotional symptoms score, the 2 X 2 ANOVA showed a main effect of the Group (F_1, 107_ = 7.488, *p* = 0.007) with higher score reported by CL (M = 3.03, SD = 2.01) rather than HC parents (M = 2.11, SD = 1.90), a main effect of the Period (F_1, 107_ = 23.080, *p* < 0.001) with higher score reported during the pandemic (M = 2.98, SD = 2.20) rather than before (M = 2.19, SD = 1.92) and no interaction effect. Regarding the parent-reported child’s hyperactivity-inattention score, the 2 X 2 ANOVA showed a main effect of the Period (F_1, 107_ = 7.830, *p* = 0.007) with higher score reported during the pandemic (M = 3.32, SD = 2.21) rather than before (M = 2.80, SD = 2.18), and the interaction Group X Period (F_1, 107_ = 10.741, *p* = 0.001). Particularly, posy-hoc analysis comparisons showed that before the pandemic, but not during (*p* = 0.999), HC parents reported that their children were less hyperactive-inattentive compared with the child of the CL group (*p* = 0.042). In addition, HC parents, but not CL parents (*p* = 0.986), reported a significant increase of hyperactivity-inattention for their child during the pandemic (*p* < 0.001). Results are presented in Table 3 and Fig. 1.

**Table 3:** Children’s emotional symptoms and hyperactivity-inattention difficulties reported by their parents before and during the COVID-19 pandemic

	Clinical group		Healthy control group		
	Before pandemic	During pandemic	Before pandemic	During pandemic	
*Children’s difficulties*	*M* ± *SD*	*M* ± *SD*	*M* ± *SD*	*M* ± *SD*	Average
Emotional symptoms	2.66 ± 2.34	3.46 ± 2.10	1.72 ± 1.54	2.51 ± 1.93	2.59 ± 1.97
Hyperactivity-inattention	3.39 ± 2.06	3.30 ± 2.81	2.21 ± 2.00	3.34 ± 2.44	3.06 ± 2.33

Finally, we run a series of linear regression to measure the association of parental anxiety and depression, COVID-19 related anxiety, parent–child difficult interaction and having or not having a child with PRD on parental perception of children’s emotional and hyperactivity-inattention symptoms. We found that anxiety, difficult parent–child interaction and having a child with PRD were significant predictors of parent perception of child’s emotional symptoms, with 37.6% explained variance (Table 4). As regards hyperactivity-inattention, parent–child difficult interaction and depressive scores predicted child’s hyperactivity-inattention, with 48.0% explained variance (Table 5).

**Table 4:** Regression analysis for emotional symptoms (dependent variable)

R = 627; R^2^ = 393; Adjusted R^2^ = .376 F (3,108) = 22.703; *p* < .001	B	SE	β	t	*p*
Anxiety	.195	.037	.424	5.268	.000
Parent-child difficult interaction	.063	.018	.285	3.538	.001
Having a child with PRD	.921	.312	.224	2.949	.004

**Table 5:** Regression analysis for hyperactivity-inattention (dependent variable)

R = 700; R^2^ = 490; Adjusted R^2^ = .480 F (2,108) = 50.920; *p* < .001	B	SE	β	t	*p*
Parent-child difficult interaction	.137	.021	.487	6.619	.000
Depression	.242	.049	.366	4.966	.000

## Discussion

PRDs affect not only children but also their entire family, with repercussions on their practical and psychological demands (Torres-Made et al., 2020), which could lead to worse psychological outcomes in this vulnerable population, especially during this critical COVID-19 period. In fact, the recent pandemic is affecting family interactions (Durcan et al., 2021), upsetting children’s environment and daily routine (Masi et al., 2021; Morelli et al., 2021) and could be a threat to the health status of individuals with a previous disease (Bramanti et al., 2021).

The present study aimed to explore this topic, also considering the lack of studies on the impact of COVID-19 pandemic on children with PRDs and their parents.

The first purpose was to detect differences in parents’ psychological adjustments (in terms of anxiety, depression, COVID-19 related anxiety, and parent–child difficult interactions) according to whether children have PRDs or not. Contrary to our expectations, results showed that there were no differences among the two groups in any of the studied variables. These results are partially consistent with a study of Durcan and co-workers (2021) who, in the group of children aged under 12 years old, found no differences between parents of children with PRDs and parents of healthy children.

The absence of differences among the two groups in our study could depend on the fact that COVID-19 pandemic represents an unexpected and critical situation, with fear of infection, for both families with severe risk of disease complications and without increased risk, causing negative psychological outcomes in both populations with and without specific medical conditions (Pınar Senkalfa et al., 2020; Sani et al., 2020).

Furthermore, it might be hypothesized that parents of healthy children, unlike parents of children with a chronic condition, usually had not experienced fear of disease before; hence, they could have high psychological distress during the pandemic, due to the health threat caused by the COVID-19 outbreak. Moreover, it is possible that having to face an autoimmune disease, such as PRDs, could lead parents to develop more adaptive responses to stressors and traumatic events, to deal with the chronicity of their children’s illness, functioning as a protective factor for psychological maladjustment (Ciaffi et al., 2020; Evers et al., 2011). However, our data do not allow us to reach a conclusion about this hypothesis, as further research with a longitudinal design is required.

A second aim of the present study was to compare children’s psychological symptoms (i.e., emotional problems and hyperactivity) reported by parents in both CL and HC groups, during and before COVID-19 outbreak. We did not formulate hypotheses in the direction of these differences, given the inconsistency of previous research. Findings showed that parents of children with PRDs reported statistically significant higher levels of both children’s emotional problems and hyperactivity before COVID-19 pandemic, compared to HC children. During COVID-19 pandemic, emotional symptoms increased for both the groups, with CL parents reporting higher levels of children’s emotional problems compared to HC group. As for the hyperactivity-inattention symptoms, only the parents of healthy children reported an increase of hyperactivity symptoms during COVID-19 outbreak. It is possible that COVID-19 restriction and the absence of social activities and friends, had a great emotional impact on both children with PRDs and healthy children (Christner et al., 2021). In line with our findings, studies conducted before COVID-19 emergency highlighted higher emotional and behavioral difficulties in children with PRDs than in healthy children (LeBovidge et al., 2003; Noll et al., 2000); these differences could depend on some disease-related factors, as disease state, pain, activity restriction, side effects of therapy and fatigue, that affects children with PRDs, making them more emotionally vulnerable (Margetić et al., 2005; Feinstein et al., 2011; Pinquart & Shen, 2011). However, factors related to the disease are not the only ones influencing their emotional and behavioral difficulties. In fact, as suggested by the disability-stress-coping model (Wallander et al., 1989), chronic pediatric illness, such as PRDs, constitutes a continuous chronic event for children that exposes them to negative life events, while intrapersonal factors (as problem-solving skills), environmental factors (as family resources) and stress processing factors (as coping and emotional regulation strategies) constitute their mechanisms of compensation against negative and stressful events (Huygen et al., 2000; Memari et al., 2016), as COVID-19 could be considered.

Despite that, differently from HC parents, CL parents did not report an increase of hyperactivity/inattention symptoms in their children during the COVID-19 emergency. It is possible that children with PRDs, due to their medical conditions, used to spend periods at home during their lives and therefore, during the COVID-19 emergency, they have not been affected at the behavioral level by the restrictive confinement measures, as happened to healthy children. However, the social isolation caused by such containment measures may have led to greater emotional difficulties as compared to pre-pandemic period, both in children with PRDs and in healthy children.

Despite the high number of studies on the psychological effects of COVID-19 among children (e.g., Jiao et al., 2020), to our knowledge none of them investigated emotional and behavioral difficulties of children with PRDs during the current pandemic. Some studies with other clinical pediatric populations, as children with developmental disorders, found increased levels of children’s emotional and behavioral difficulties (Conti et al., 2020). Studies among healthy children detected negative psychological outcomes during COVID-19 emergency (Babore et al., 2021a, b; Wang et al., 2020). Some measures implemented to avoid the spread of the virus (such as social isolation, the closure of schools and the disruption of daily routine) could negatively affect children’s mental health (Trumello et al., 2021). At the same time, changes in the daily disease management routine caused by COVID-19 lockdown might have potentially affected the clinical course of PRDs, bringing fewer physical activity and increased watching TV hours and/or video games playing time (Naddei et al., 2021).

Regarding the third aim, we found that higher levels of parental anxiety, worse parent–child interaction and having a child with PRDs were associated with greater children’s emotional difficulties.

Parental distress could affect children’s wellbeing, influencing parental sensitivity to children needs and parent–child interaction, leading to greater children’s emotional and behavioral problems (Babore et al., 2021a, b; Coté et al., 2009). Some studies highlighted the role of parental anxiety on children’s emotional problems during COVID-19 emergency (Khoury et al., 2021; Dubois-Comtois et al., 2021; Hanetz-Gamliel et al., 2021).

It could not be excluded that having a child with PRDs could make parents more vulnerable to negative psychological outcomes, such as anxiety, depression, and stress, due to the multiple challenges that they have to manage, as physical, practical and psychosocial implications of the disease, resulting in greater emotional and behavioral difficulties for their children (Timko et al., 1992; Kietz, 2004; Ryan et al., 2010; Yuwen et al., 2017). However, further longitudinal studies should address this issue to explore cause-and-effects relationships. Besides, our findings highlighted an association of children’s hyperactivity symptoms with parent–child difficult interaction and higher levels of parental depression. A few studies highlighted that parental depression affects children’s behavioral difficulties, but not emotional problems (Harris et al., 1991; Middleton et al., 2009). Some studies showed that parental depression influences parental practice and parent–child interaction (De Giacomo et al., 2021; Lovejoy et al., 2000; Middleton et al., 2009) leading to increased behavioral problems in the offspring. However, previous studies highlighted a bidirectional relationship between parental depression and children’s externalizing problems (Nicholson et al., 2011; Shaw et al., 2009). It is possible that parental depression leads to less sensitive parenting and higher rates of irritability, causing higher hyperactivity and conduct problems in children. At the same time, conduct problems may contribute to reinforce depressive symptoms in parents. Hence, we cannot exclude that the negative effects produced by the COVID-19 social restrictions on children’s behavioral symptoms may have had a negative impact on parents’ depressive levels. Unfortunately, the cross-sectional nature of our study prevents us to identify cause-and-effect relationships. We have to mention some further limitations of the present study. First of all, our findings are based on parents’ self-report and also the measures concerning children’s difficulties were assessed by the parents. This means that we have not assessed the children’s experience from their own point of view. A recent study found a high consistency between mothers’ and children’s perceptions of children’s psychological adjustment (Babore et al., 2021a); however, future research could further explore if the parents’ perception matches with the actual psychological status of the children. Another limitation regards the unbalanced representation of both parents as most participants comprised mothers. Also, according to the abovementioned cross-sectional nature of the study, all the data were collected during the COVID-19 emergency, although we requested parents to report children’s psychological status before the pandemic, and this does not allow us to exclude a retrospective bias. To address these issues, longitudinal and multi-informant studies are needed to evaluate risk factors involved in psychological adjustment during the COVID-19 outbreak, especially in children with chronic diseases and their parents. Moreover, larger population-based studies are necessary to better characterize the parent–child relationship in PRDs.

Despite these limits, our study has the merit to highlight the importance to target the children in relation to their context, when approaching the psychological aspects of PRDs.

## Data Availability

The datasets analyzed during the current study are available from the corresponding author on reasonable request.
